# Graphs and Matroids Weighted in a Bounded Incline Algebra

**DOI:** 10.1155/2014/912715

**Published:** 2014-07-14

**Authors:** Ling-Xia Lu, Bei Zhang

**Affiliations:** ^1^School of Mathematics and Science, Shijiazhuang University of Economics, Shijiazhuang 050031, China; ^2^School of Science, Hebei University of Science and Technology, Shijiazhuang 050018, China

## Abstract

Firstly, for a graph weighted in a bounded incline algebra (or called a dioid), a longest path problem (LPP, for short) is presented, which can be considered the uniform approach to the famous shortest path problem, the widest path problem, and the most reliable path problem. The solutions for LPP and related algorithms are given. Secondly, for a matroid weighted in a linear matroid, the maximum independent set problem is studied.

## 1. Introduction

In graph theory, the famous shortest path problem (SPP, for short) is the problem of finding a path between two vertices in a weighted graph such that the sum of the weights of its constituent edges is minimized [1]. An example is finding the quickest way to get from one location to another on a road map. In this case, the vertices represent locations and the edges represent segments of road and are weighted by the time needed to travel that segment.

If we assume the weighted function to be nonnegative, then the related algebraic foundation of SPP is the semiring ([0, +*∞*], min⁡, +). Therein, we use the operation “+” to compute the length of paths and use the operation “min” to find the least one. For the widest path problem (WPP, for short) or called the greatest capacity problem (GCP, for short), the related algebraic foundation is the semiring ([0, +*∞*], max⁡, min⁡). Accordingly, we use the operation “min” to compute the capacities and use the operation “max” to find the greatest one. For the most reliable path problem (MRPP, for short), the related algebraic foundation is the semiring ([0,1], max⁡, ×). Accordingly, we use the operation “×” to compute the reliability of paths and use the operation “max” to find the greatest one. There are many other classical problems using various semirings in graph theory [2].

For both ([0, +*∞*], min⁡, +) for SPP and ([0, +*∞*], max⁡, min⁡) for WPP as well as the corresponding algorithms, the value “+*∞*” is used to act as the weight of artificial edges between vertex pairs with no edge. For these reasons, SPP, WPP, and MRPP (and other potential problems) can be put into a more generalized setting: the algebraic path problem [2]. The first aim of this paper is to unify SPP, WPP, WPP, and other path problems into graphs weighted in an idempotent semiring (also known as a dioid) [3]. We shall give a unified approach to find the shortest path, the widest path, and the most reliable path as well as their length.

In 1935, Whitney introduced matroids as a generalization of both graphs and linear independence in vector spaces [4]. It is well known that matroids play an important role in applied mathematics, especially in optimal theory, which are precisely the structures for the maximum independent set problem (MISP, for short) which the very simple and efficient greedy algorithm works [5]. The second aim of this paper is to study matroids weighted in a linear dioid and the related MISP.

## 2. Semirings, Incline Algebras, Dioids, and Their Properties

Semirings and matrices over semirings are useful tools in diverse areas such as automata theory, design of switching circuits, graph theory, medical diagnosis, Markov chains, informational systems, complex systems modeling, decision-making theory, dynamical programming, control theory, nervous system, probable reasoning, psychological measurement, and clustering [3, 6].

**Definition 1 (see [3]).**

A (2,2)-type algebra (*K*, ⊕, ⊗) is called a semiring if

(K1) both (*K*, ⊕) and (*K*, ⊗) are semigroups;
(K2) ⊕ is commutative; that is, *a* ⊕ *b* = *b* ⊕ *a* for all *a*, *b* ∈ *K*;
(K3) ⊗ is distributive over ⊕; that is *a* ⊗ (*b* ⊕ *c*) = (*a* ⊗ *b*)⊕(*a* ⊗ *c*); (*b* ⊕ *c*) ⊗ *a* = (*b* ⊗ *a*)⊕(*c* ⊗ *a*) for all *a*, *b*, *c* ∈ *K*.

We call a semiring (*K*, ⊕, ⊗) preunital if there is a special element 1 ∈ *K* such that (*K*, ⊗, 1) is a monoid; that is, 1 ⊗ *x* = *x* = *x* ⊗ 1 for every *x* ∈ *K*.

**Proposition 2 .**

For every preunital semiring (*K*, ⊕, ⊗, 1), the following conditions are equivalent:

(K4) 1 is absorbing with respect to the operation ⊕; that is, 1 ⊕ *x* = 1 for every *x* ∈ *K*;
(K4′) *x* ⊕ (*x* ⊗ *y*) = *x* and (*x* ⊗ *y*) ⊕ *y* = *y* for all *x*, *y* ∈ *K*.

**Proof**

(K4)⇒(K4′): *x* ⊕ (*x* ⊗ *y*) = (*x* ⊗ 1)⊕(*x* ⊗ *y*) = *x* ⊗ (1 ⊕ *y*) = *x* ⊗ 1 = *x*. Similarly, (*x* ⊗ *y*) ⊕ *y* = *y*.

(K4′)⇒(K4): 1 ⊕ *x* = 1 ⊕ (1 ⊗ *x*) = 1.

A preunital semiring with condition (K4) is called a unital semiring. A semiring (*K*, ⊕, ⊗) is called idempotent if ⊕ is idempotent; that is, *a* ⊕ *a* = *a* for all *a* ∈ *K*. An idempotent semiring with the condition (K4′) is called an incline algebra [6].

**Proposition 3 .**

Suppose that (*K*, ⊕, ⊗) is a unital semiring and 0 is an element in *K*. Then the following conditions are equivalent:

(K5) 0 is absorbing with respect to the operation ⊗; that is, 0 ⊗ *x* = 0 = *x* ⊗ 0 for every *x* ∈ *K*;
(K5′) (*K*, ⊕, 0) is a monoid; that is, 0 ⊕ *x* = *x* for every *x* ∈ *K*.

**Proof**

(K5)⇒(K5′): by (K4′), 0 ⊕ *x* = (0 ⊗ *x*) ⊕ *x* = *x*.

(K5′)⇒(K5): by (K4′), 0 ⊗ *x* = 0 ⊕ (0 ⊗ *x*) = 0.

An idempotent and unital semiring (*K*, ⊕, ⊗) with condition (K5) is called a dioid; that is, a dioid is an incline with (K4′) and (K5′), which is called a bounded incline algebra [6].

In every dioid (*K*, ⊕, ⊗, 0, 1), we define *x*⪯*y* iff *x* ⊕ *y* = *y*. Then ⪯ is a partial order on *K* and (*K*, ⊕) is a bounded join semilattice. Clearly, 0 is the bottom element and 1 is the top element, so is the name bounded incline.

**Proposition 4 (see [7]).**

If *a*⪯*b*, *c*⪯*d*, then *a* ⊕ *c*⪯*b* ⊕ *d* and *a* ⊗ *c*⪯*b* ⊗ *d*.

**Proof**

Since *a*⪯*b*, *c*⪯*d*, we have *a* ⊕ *b* = *b*, *c* ⊕ *d* = *d*:

1. (*a* ⊕ *c*)⊕(*b* ⊕ *c*) = (*a* ⊕ *b*)⊕(*c* ⊕ *d*) = *b* ⊕ *d* and *a* ⊕ *c*⪯*b* ⊕ *d*;
2. (*a* ⊗ *c*)⊕(*b* ⊗ *c*) = (*a* ⊕ *b*) ⊗ *c* = *b* ⊗ *c* and then *a* ⊗ *c*⪯*b* ⊗ *c*. Similarly, *b* ⊗ *c*⪯*b* ⊗ *d*. Hence *a* ⊗ *c*⪯*b* ⊗ *d*.

**Example 5 (classical examples).**

(1) ([0, +*∞*], min⁡, +, +*∞*, 0) is a dioid, which is an algebraic model for SPP. The partial order ⪯ defined above is dual to the usual one ≤. For explicit, *a*⪯*b* iff *b* ≤ *a* in usual meaning.

(2) ([0, +*∞*], max⁡, min⁡, 0, +*∞*) is a dioid, which is an algebraic model for WPP. The partial order ⪯ defined above is the same as the usual one ≤.

(3) ([0,1], max⁡, ×, 0,1) is a dioid, which is an algebraic model for MRPP. The partial order ⪯ defined above is the same as the usual one ≤.

**Example 6 (other examples).**

Consider

1. ([0, +*∞*], min⁡, max⁡, +*∞*, 0);
2. ([0,1], min⁡, max⁡, 1,0);
3. ([0,1], max⁡, min⁡, 0,1);
4. ([1, +*∞*], min⁡, ×, +*∞*, 1).

Let *A* be an (*m* × *n*)-matrix and let *B* be an (*n* × *l*)-matrix over a semiring (*K*, ⊕, ⊗). Define *A*∘*B* = (*p*_*ij*_)_*m*×*l*_ by *p*_*ij*_ = ⊕_*k*=1_^*n*^(*a*_*ik*_ ⊗ *a*_*kj*_).

**Proposition 7 .**

Let *A*_*kl*_, *B*_*lm*_, *C*_*mn*_ be three matrices. Then (*A*_*kl*_∘*B*_*lm*_)∘*C*_*mn*_ = *A*_*kl*_∘(*B*_*lm*_∘*C*_*mn*_).

**Proof**

Let *A*_*kl*_∘*B*_*lm*_ = (*p*_*ij*_)_*km*_, *B*_*lm*_∘*C*_*mn*_ = (*q*_*ij*_)_*lm*_ and (*A*_*kl*_∘*B*_*lm*_)∘*C*_*mn*_ = (*u*_*ij*_)_*ln*_, *A*_*kl*_∘(*B*_*lm*_∘*C*_*mn*_) = (*v*_*ij*_)_*ln*_. Then

(1) uij=⊕s=1m(pis⊗csj)=⊕s=1m((⊕t=1l(ait⊗bts))⊗csj)=⊕s=1m⊕t=1l[(ait⊗bts)⊗csj]=⊕t=1l⊕s=1m[ait⊗(bts⊗csj)]=⊕t=1l[ait⊗(⊕s=1m(bts⊗csj))]=⊕t=1l(ait⊗qtj)=vij.

## 3. Graphs Weighted in a Dioid and the Longest Path Problem

For the dioid ([0, +*∞*], min⁡, +, +*∞*, 0) in SPP, since the partial order ⪯ is dual to the usual one ≤, the SPP in the dioid situation comes to be a longest path problem (LPP for short). In this section, we will study the LPP for graphs weighted in a dioid, which can be considered a unified approach for SPP, WPP, and MRPP (and so on).

Let *G* be a graph weighted in a dioid (*K*, ⊕, ⊗, 0, 1). For two vertices *i*, *j*, let *P*_*ij*_ denote the set of paths from *i* to *j*. For *p* ∈ *P*_*ij*_, *w*_*p*_ = ⊗_*e*∈*p*_ *w*(*e*) is called the length of the path *p*. Since ⊕ is idempotent, *p*_*ij*_ = ⊕_*p*∈*P*_*ij*__ *w*_*p*_ is the longest path length from *v*_*i*_ to *v*_*j*_. For the weighted graph *G*, define a matrix *A* = (*a*_*ij*_)_*n*×*n*_ by the following:

1. for *i* ≠ *j*, if there are some paths from *v*_*i*_ to *v*_*j*_, then put *a*_*ij*_ as the maximal weight of all parallel edges from *v*_*i*_ to *v*_*j*_; if there is no path from *v*_*i*_ to *v*_*j*_, then put *a*_*ij*_ = 0;
2. for every *i*, put *a*_*ii*_ = 1.

For any 1 ≤ *i*, *j* ≤ *n*, define *a*_*ij*_^(1)^ = *a*_*ij*_ and *a*_*ij*_^(*m*+1)^ = ⊕_*l*=1_^*n*^(*a*_*il*_^(*m*)^ ⊗ *a*_*lj*_^(1)^) (*m* = 1,2, 3,…).

**Proposition 8 .**

(1) *a*_*ij*_^(*m*)^ is the longest *m*-step path from *v*_*i*_ to *v*_*j*_.

(2) For any *m* = 0,1, 2, ,…, then *a*_*ij*_^(*m*)^⪯*a*_*ij*_^(*m*+1)^.

(3) *a*_*ij*_^(*m*)^ = *a*_*ij*_^(*n*)^ for all *m* ≥ *n*.

**Proof**

(1) We use the induction to prove this result. For *k* = 1, the result holds. Suppose that the result holds for *k* = *m*. For *k* = *m* + 1, *a*_*ij*_^(*m*+1)^ = ⊕_*k*=1_^*n*^(*a*_*ik*_^(*m*)^ ⊗ *a*_*kj*_^(1)^). Let *e*_1_, *e*_2_,…, *e*_*m*_, *e*_*m*+1_ be an (*m* + 1)-step path from *v*_*i*_ to *v*_*j*_. Then *e*_1_, *e*_2_,…, *e*_*m*_ is an *m*-step path from *v*_*i*_ to *v*_*k*_, where *v*_*k*_ is the end vertex of *e*_*m*_; and *e*_*m*+1_ is an edge from *v*_*k*_ to *v*_*j*_ and *a*_*kj*_^(1)^⪰*w*(*e*_*m*+1_). Then *a*_*ik*_^*m*^⪰*w*(*e*_1_) ⊗ *w*(*e*_2_)⊗⋯⊗*w*(*e*_*m*_) and *a*_*ij*_^(*m*+1)^⪰*a*_*ik*_^(*m*)^ ⊗ *a*_*kj*_^(1)^⪰*w*(*e*_1_) ⊗ *w*(*e*_2_) ⊗ ⋯⊗*w*(*e*_*m*_) ⊗ *w*(*e*_*m*+1_). This completes the proof.

(2) *a*_*ij*_^(*m*+1)^ = ⊕_*l*=1_^*n*^(*a*_*il*_^(*m*)^ ⊗ *a*_*lj*_^(1)^)⪰*a*_*ij*_^(*m*)^ ⊗ *a*_*jj*_^(1)^ = *a*_*ij*_^(*m*)^ ⊗ 1 = *a*_*ij*_^(*m*)^.

(3) Suppose that *m* ≥ *n*. By (2), we have *a*_*ij*_^(*m*)^⪰*a*_*ij*_^(*n*)^. By (1), *a*_*ij*_^(*m*)^ is the longest *m*-step path from *v*_*i*_ to *v*_*j*_. Suppose that the related path of *a*_*ij*_^(*m*)^ is *p* = {*v*_*l*_1__, *v*_*l*_2__,…, *v*_*l*_*m*__}, since there is at most *n* vertices in *G* and there are some common points in *p*. Suppose that *v*_*l*_*i*__ and *v*_*l*_*j*__ (let *l*_*i*_ ≤ *l*_*j*_) are the same point. In order to make *a*_*ij*_^(*m*)^ the longest path, it must hold that *v*_*l*_ = *v*_*l*_*i*__ = *v*_*l*_*j*__ for all *l*_*i*_ ≤ *l* ≤ *l*_*j*_. Then the length *a*_*ij*_^(*m*)^ is equal to the length of a path from *v*_*i*_ to *v*_*j*_ with no common point (with at most *n* vertices). Hence *a*_*ij*_^(*m*)^⪯*a*_*ij*_^(*n*)^ since *a*_*ij*_^(*n*)^ is the longest *n*-step path from *v*_*i*_ to *v*_*j*_.

We now present two algorithms to compute the longest path length and the corresponding longest path.

**Algorithm 9 .**

To find the longest path length from a vertex *v*_*i*_ to another one *v*_*j*_,

 input: *A*_*ij*_ and *i*, *j*;
 output: the longest path length from *v*_*i*_ to *v*_*j*_;
 (1) *a*_*il*_^(1)^ = *a*_*il*_ (*l* = 1,2,…, *n*); *a*_*hj*_^(1)^ = *a*_*hj*_, (*h* = 1,2,…, *n*);
 (2) for *m* = 1 to *n* do. Put *a*_*ij*_^(*m*+1)^ = ⊕_*l*=1_^*n*^(*a*_*il*_^(*m*)^ ⊗ *a*_*lj*_^(1)^). If *a*_*ij*_^(*m*+1)^ = *a*_*ij*_^(*m*)^, then print “the longest path length is *a*_*ij*_^(*m*)^.”

**Algorithm 10 .**

Suppose that the longest path length from *v*_*i*_ to *v*_*j*_ is *a*_*ij*_^(*m*)^. To find the related longest path,

 input: *a*_*il*_^(1)^, *a*_*il*_^(2)^,…, *a*_*il*_^(*m*)^ for *l* ∈ {1,2 …, *n*};
 output: the longest path from *v*_*i*_ to *v*_*j*_;
 (1)  *p* = {*v*_*j*_};
 (2) for *h* = *m* to 1 do. Find *l* ∈ {1,2,…, *n*} such that *a*_*ij*_^(*h*)^ = *a*_*il*_^(*h*−1)^ ⊗ *a*_*lj*_^(1)^;
 (3) *p* ← {*v*_*l*_} ∪ *p*;
 (4) print “*p*”.

## 4. Matroids Weighted in a Linear Dioid and the Maximum Independent Set Problem

For a classical graph *G* = (*E*, *V*), let *I*(*G*) = {*I*⊆*E*∣*I* has no circuit}. Then (*E*, *I*(*G*)) is a matroid; that is,

(I1) *∅* ∈ *I*;
(I2) *A*⊆*B* ∈ *I* implies *A* ∈ *I*;
(I3) for *A*, *B* ∈ *I*, if |*A* | <|*B*|, then there exists *e* ∈ *B* − *A* such that *A* ∪ *e* ∈ *I*.

Similar to weighted graph, matroids also play an important role in mathematics, especially in applied mathematics, which are precisely the structures for which the very simple and efficient greedy algorithm works [5].

In this section, we will study matroids weighted in a linear dioid (notice that all the examples in Examples 5 and 6 are linear) and the maximum independent set problem.

We suppose that (*K*, ⊕, ⊗, 1, 0) is a linear dioid. Let *E* be a finite set and *M* = (*E*, *I*) a matroid weighted in *K* with *w* : *E* → *K* being the weighted function.

In the optimization theory, the maximum independent set problem (MISP, for short) is to find an independent subset *J* ∈ *I*(*M*) such that *w*(*J*) = max⁡{*w*(*I*)∣*I* ∈ *I*(*M*)}. We will use the famous greedy algorithm to deal with this problem.

The greedy algorithm:

1. labeling *E* = {*e*_1_, *e*_2_,…, *e*_*m*_} such that *w*(*e*_1_)⪰*w*(*e*_2_)⪰⋯⪰(*e*_*m*_);
2. *J* = :*∅*;
3. for *i* = 1 to *n* do, if *J* ∪ *e*_*i*_ ∈ *I*(*M*), then *J* ← *J* ∪ *e*_*i*_.

**Proposition 11 .**

The greedy algorithm has an optimal solution.

**Proof**

Suppose that *J* is a solution of GA. Then *J* ∈ *I*(*M*). For any *J*′ ∈ *I*(*M*), we need to show that *w*(*J*)⪰*w*(*J*′). Suppose that *J* = {*e*_*i*_1__, *e*_*i*_2__,…, *e*_*i*_*k*__} and *J*′ = {*e*_*j*_1__, *e*_*j*_2__,…, *e*_*j*_*l*__} with *w*(*e*_*i*_1__)⪰*w*(*e*_*i*_2__)⪰⋯⪰*w*(*e*_*i*_*k*__) and *w*(*e*_*j*_1__)⪰*w*(*e*_*j*_2__)⪰⋯⪰*w*(*e*_*j*_*l*__). On one hand, by the algorithm, we have *i*_*k*_ ≥ *j*_*l*_; that is, |*J* | ≥|*J*′|. On the other hand, if *w*(*J*′)⪰*w*(*J*), then there exits a least integral number *s* > 0 such that *w*(*e*_*j*_*s*__)⪰*w*(*w*_*i*_*s*__). Put *I* = {*e*_*i*_1__, *e*_*i*_2__,…, *e*_*i*_*s*__} and *X* = *I* ∪ {*e*_*j*_1__, *e*_*j*_2__,…, *e*_*j*_*s*__}. We have *I* ∈ *I*(*M*∣*X*). By the minimality of *s*, for any *t* ∈ {1,2,…, *s*}, if *e*_*j*_*t*__ ∉ *I*, then *I* ∪ *e*_*j*_*t*__ ∉ *I*(*M*∣*X*). Hence *r*(*M*∣*X*) = *s* − 1, which is contradicting with {*e*_*j*_1__, *e*_*j*_2__,…, *e*_*j*_*s*__} ∈ *I*(*M*∣*X*). For a summary, we have *w*(*J*′)⪯*w*(*J*). This completes the proof.

**Proposition 12 .**

Suppose that *M* = (*E*, *I*) is a matroid and *K* is a linear dioid. Let *P*(*M*) = {*x* ∈ *K* ^*E*^∣∀ *A*⊆*E*, *x*(*A*)⪯*r*(*A*)}. Then the set of maximal points is precisely the characteristic vectors of all independent sets in *M*.

**Proof**

Suppose that *x*′ is a maximal point of *P*(*M*). Then there exists a vector *c* ∈ *K* ^*E*^ such that the following optimal problem has a unique solution.

*Problem*. max⁡{*w* · *x*∣*x* ∈ *P*(*M*)}, where *w* : *E* → *K* is a weighted function.

By greedy algorithm, the solution of this problem has the form *x*_*J*_ for some independent set *J*. Then *x*′ = *x*_*J*_. On the other hand, if *J* ∈ *I*(*M*), then it is easily seen that *x*_*J*_ is a maximal point of *P*(*M*).

In [8, 9], for a complete lattice *L*, Shi introduced an approach to fuzzification of matroids, namely, an *L*-fuzzifying matroid, which are successfully characterized by a kind of fuzzy rank functions. Consequently, the corresponding axioms of bases and circuits, dependent sets, and closure operators are established, by which *L*-fuzzifying matroids are also equivalently characterized [10–12].

Of course, for a complete lattice *L*, we know that (*L*, ∨, ∧, 0,1) is a special dioid. So, a natural question arises: Can we generalize the truth value table *L* of Shi's *L*-fuzzifying matroid to a dioid? We here try a first attempt to give a positive answer.

**Definition 13 .**

Suppose that (*K*, ⊕, ⊗, 0, 1) is a dioid and let *E* be a finite set. A map *I* : 2^*E*^ → *K* is a map satisfying the followin:

 (FI1)  *I*(*∅*) = 1;
 (FI2) if *A*⊆*B*, then *I*(*B*)⪯*I*(*A*);
 (FI3) if |*A* | <|*B*|, then *I*(*A*) ⊗ *I*(*B*)⪯⊕_*e*∈*B*−*A*_ *I*(*A* ∪ *e*).

The pair (*E*, *I*) is called a *K*-fuzzifying matroid.

By [12], we know that a graph weighted in the unit interval [0,1] induces a [0,1]-fuzzifying matroid in a natural way. For a dioid *K*, we have the similar results.

**Proposition 14 .**

Suppose that *G* = (*E*, *V*, *w*) is a weighed graph in a dioid (*K*, ⊕, ⊗). Define *J*_*G*_ : 2^*E*^ → *K* by *I*_*G*_(*A*) = ⊗_*x*∈*A*_ *x* if *A* is independent and 0 otherwise. Then (*E*, *I*) is a *K*-fuzzifying matroid.

**Proof**

The proof is a routine.
